# Robotic-assisted unicompartmental knee arthroplasty: a review

**DOI:** 10.1186/s42836-021-00071-x

**Published:** 2021-05-02

**Authors:** Pei Liu, Fei-fan Lu, Guo-jie Liu, Xiao-hong Mu, Yong-qiang Sun, Qi-dong Zhang, Wei-guo Wang, Wan-shou Guo

**Affiliations:** 1 Department of Adult Joint Reconstruction, Henan Luoyang Orthopaedic Hospital (Henan Provincial Orthopaedic Hospital), Yongping Road, Zhengdong New District, Zhengzhou, China; 2 China-Japan Friendship School of Clinical Medicine, Peking University, Yinghuadong Road, Chaoyang District, Beijing, China; 3 Department Orthopedics 4, Beijing University of Chinese Medicine, Dongzhimen Hospital, Beijing, China; 4 Department of Orthopaedic Surgery, Beijing Key Lab Immune-Mediated Inflammatory Diseases, China-Japan Friendship Hospital, No. 2, Yinghuadong Road, Chaoyang District, Beijing, 100029 China

**Keywords:** Robotic assisted surgery, Robotic, Unicompartmental knee arthroplasty

## Abstract

**Purpose:**

Presented here is an up-to-date review concerning robotic-assisted unicompartmental knee arthroplasty (rUKA), including its rationale, operative system, pros and cons.

**Methods:**

We did a systematic research in electronic databases, including PubMed, Cochrane Library, Web of Science, and Embase up to March 30, 2020 to retrieve literature pertaining to rUKA. The search strategies “(robotic* AND knee arthroplasty OR knee replacement)” and “(knee arthroplasty OR knee replacement NOT total)” were used. Studies describing rUKA and clinical trials, dry bone or cadaveric researches regarding technologies, positioning, alignment, function, or survivorship of implants were included in this review. All retrieved studies were first browsed for eligibility on the basis of title and abstract, and the selected studies were further evaluated by reading full text for final inclusion.

**Results:**

Robotic-assisted technology has been found to increase the accuracy of bone preparation and implant placement, reduce technical variability and outliers, and enhance reproduction of limb alignment. Additionally, early clinical outcomes were excellent, but mid-term follow-up showed no superiority in component survivorship. The potential drawbacks of the robotic-assisted technology include relatively-low time- and cost-effectiveness, development of some rUKA-related complications, and lack of support by high-quality literature.

**Conclusion:**

This review shows that rUKA can decrease the number of outliers concerning the optimal implant positioning and limb alignment. However, due to absence of extensive studies on clinical outcomes and long-term results, it remains unclear whether the improved component positioning translates to better clinical outcomes or long-term survivorship of the implant. Nevertheless, since an accurate implant position is presumably beneficial, robotic-assisted technology is worth recommendation in UKA.

## Introduction

Unicompartmental knee arthroplasty (UKA) is a promising procedure since it preserves bone and ligaments, shortens hospital stay time, reduces postoperative morbidity, and enhances patient’s satisfaction compared with total knee arthroplasty (TKA) in the treatment of end-stage symptomatic anteromedial osteoarthritis and focal osteonecrosis of knee [1, 2]. Nonetheless, UKA is a significantly demanding technique. Up to 30% of UKAs using the standard operative technique resulted in inaccurate implantation [3]. In addition, it is challenging to achieve precise limb alignment with conventional techniques, particularly in minimally invasive procedures. Even skilled surgeons may not consistently attain accurate alignment [4]. Investigations by Keene and Cobb demonstrated that, in as many as 40% to 60% cases, outliers were over 2 degrees more than the preoperatively scheduled alignment [5, 6]. UKA was also associated with higher revision rate and lower survivorship [7–9].

Encouraged by improving clinical outcomes and survivorship due to use of more accurate placement of implants and optimal balance of soft-tissue, clinicians are putting into use more technologies, such as patient-specific cutting guides, computer navigation, and semi-customized patient-specific implants. Robotic-assisted unicompartmental knee arthroplasty (rUKA) aims to simplify procedures, maximize the accuracy of bone preparation and component positioning, reduce outliers, restore alignment as desired, and eventually improve clinical outcomes and prolong implant durability [10–13]. In the United States, 15∼20% of UKAs are performed under the assistance of robotic devices, and the rate is projected to reach over 37% in the next decade [14]. Furthermore, publications and patents related to raUKA have also been on the rise dramatically [15]. However, recent studies showed that improved precision failed to benefit function recovery, lower revision rate or protract implant survivorship [16–18]. Moreover, the problem of time- and cost-effectiveness of rUKA has yet to be resolved.

**Fig. 1:**
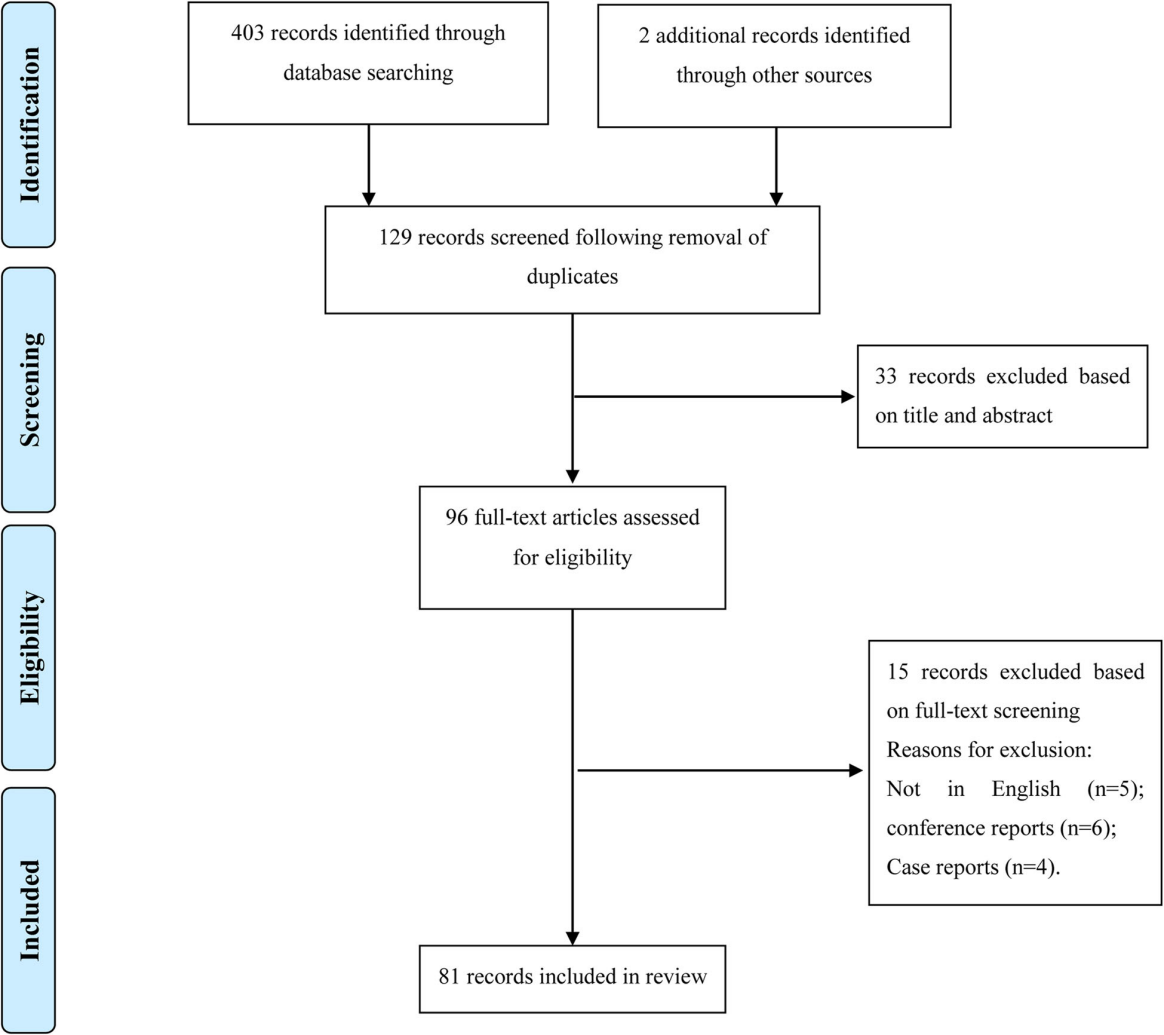
PRISMA flow diagram outlining article/abstract selection process

This review looked into the historical development and current application of rUKA and made some predictions about its future (Fig. 1).

### History of robotic-assisted surgery

The rationales of robotic-assisted surgery mainly refer to preoperative plan, intraoperative guidance, and smart remote surgical technologies [19]. The first robotic-assisted surgery was performed in neurosurgical biopsies dated back to 1985, showing that the procedure could accomplish higher precision [20]. In 1989, Davies *et al*. published their results of transurethral resection of prostate [21]. Their study confirmed that using robotics to handle soft tissue in surgery was practicable. Since then, application of medical robotic-assisted technologies have been growing worldwide. The first robotic-assisted total hip arthroplasty (THA) was reportedly performed in 1992 [22]. A robotic device for performing TKA was first described in 1993 [23], and the Acrobot assistance system used for UKA was introduced initially in 2000. In 2006, Cobb reported that the robotic-assisted technology resulted in more accurate tibiofemoral alignment in UKA [6]. To date, with the rapid development of robotic assistance technology and increasing robotic-assisted procedures performed in UKA, robotic-assisted systems have also been used in patellofemoral and bicompartmental knee arthroplasty [24, 25].

### Platforms of the technology

#### Passive, active, and semi-active robotics

According to autonomy of robotics, medical robotics fall into three types: passive, semi-active and active robotics [26]. Passive robotic systems provide recommendations for perioperative guidance of positioning, but surgical procedures have to be directly performed by the surgeon, without real robotic assistance. A typical example of passive systems is the OMNIBotics system. Active systems are capable of performing the surgery autonomously through pre-programmed algorithms and defined parameters for bone resection, and the surgeon simply controls the “shut-off” switch in emergency. A historical example is the RoboDoc system. Semi-active systems, for example the Mako system, are those in which surgical tasks are adjusted or constrained by the system, but the final execution of the operation still depends on the surgeon.

#### Image-based *vs*. imageless

A “pre-approved” execution plan before bone resection is essential for rUKA. The robotic-assisted systems are image-based or imageless, depending on preoperative and/or intraoperative mapping. Patients’ anatomical structures must be registered via mapped points on the bone with a steering tool, so that the “robot” knows the space for the cutting tools, and a poor registration will lead to reduced accuracy.

With image-based systems, the registration is associated with patient-specific digital imaging data, usually using the ipsilateral hip, knee and ankle computed tomography (CT) scan. These imaging data are stored in the robotic system to accurately identify depth of bone resection, alignment and deformities that need to be corrected, and the boundaries of bone removal. The robot then carries out the surgeons’ approved plan during procedure. Shortcomings of image-based system include added cost and radiation exposure [27]. Without image scan, imageless systems depend on the registration of the knee anatomy after surgical exposure by creating a virtual model. The surgical plan is carried out during the procedure and is updated according to the process of registration. This registration mainly depends on the accuracy of inputting data points, generally using computer navigation registration, which is easy to locate and label important landmarks. Advantages of imageless system include lower cost of the images, freedom from preoperative radiation exposure, and convenience to the patients. The possible disadvantages are lack of preoperative plan and inability to confirm the anatomic registration points.

#### Closed *vs*. open platforms

In terms of differences in compatibility, robotic systems are of two types: closed and open platforms. The closed one is designed for the implants of a single manufacturer, while the open one can adapt to the products of different companies. The open platform is designed to be used on the basis of the surgeon’s preference or patients’ requirement, but some unique features have vanished. For example, lack of design depth and biomechanical data does not allow optimization of component positioning. If coupled with an imageless system, some features and preoperative protocols are not applicable [28]. The ability of the closed platform to generate a visual image of the implant is attractive. Since prostheses need to be improved and software needs to be constantly upgraded, this “attractiveness” is both capital- and labor-intensive. The clinical effectiveness of the two platforms is still controversial. With the operative system becoming increasingly user-friendly, surgeons are in a position to decide when to use the system as desired.

### Contemporary rUKA systems

**Fig. 2:**
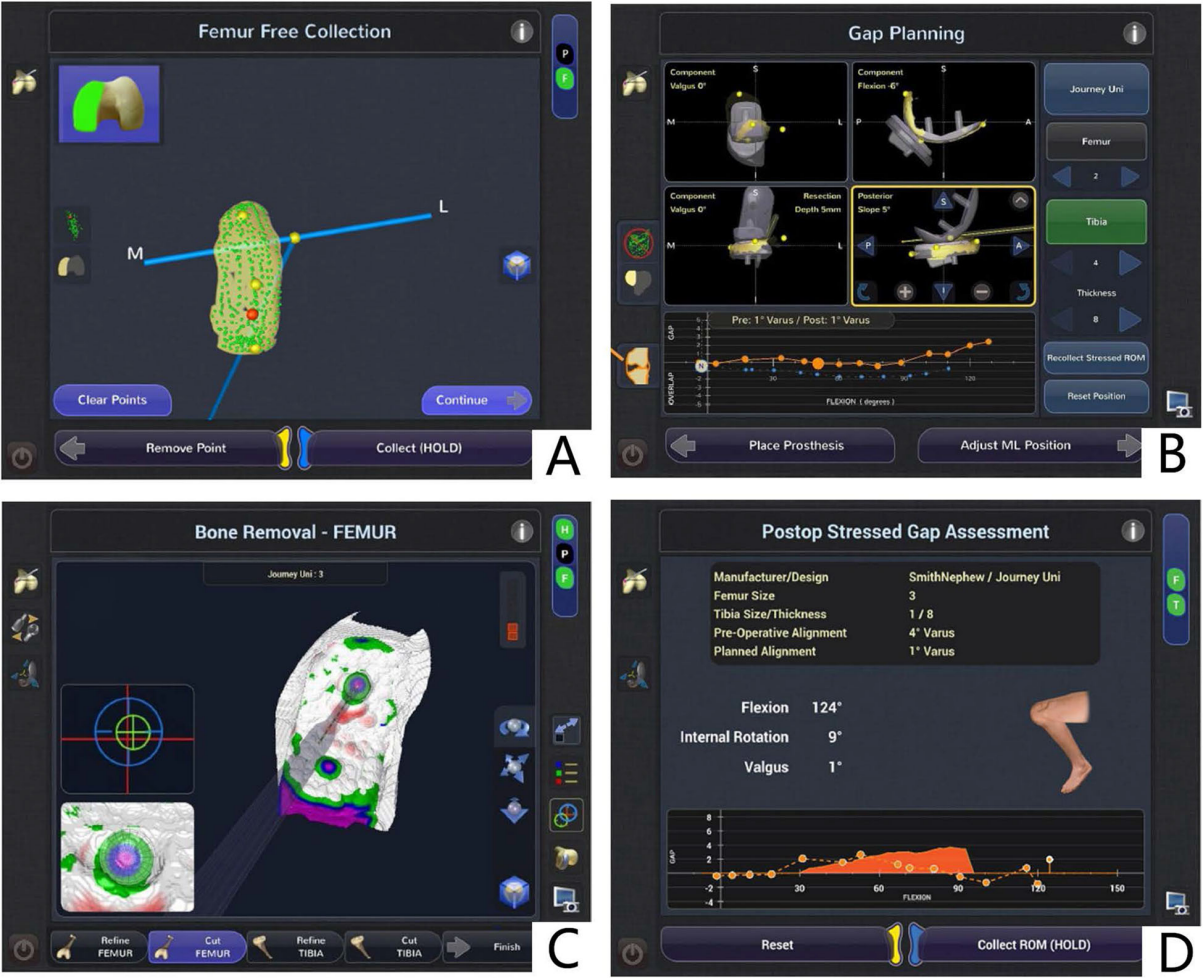
A: Surface model formation of the femur during registration. **B**: Planning screen to show predicted gaps throughout a range of flexion. **C**: Screen guidance during bone reaming showing the remaining bone to be removed. **D**: Postoperative gap assessment under stress throughout a range of flexion [29]

Most robotic-assisted systems are structurally comparable. The steps to a rUKA involve (1) establishing a specific model and designing a preoperative plan; (2) registering the model and plan based on the patient’s anatomy during pre- or/and intraoperative period; and (3) implementing the preoperative plan in the patient under the guidance of robotic assistance. The most-used robotic-assisted systems include the Acrobot (Acrobot Ltd., Elstree, London, UK), Navio (Smith and Nephew, Pittsburgh, Pennsylvania, USA), and MAKO System (MAKO Surgical Corporation, Fort Lauderdale, Florida, USA) (Fig. 2). To date, no studies compared the accuracy or outcomes of one system against the others.

The Acrobot system is the first used robotic-assisted system in UKA and a prototype to modern haptic systems. It adopts an image-based closed semi-active robotic system, which restrains the motion to a pre-defined surgical region, and thereby enables the surgeon to safely cut affected knee bones to fit UKA prosthesis precisely. Intraoperatively, a non-invasive anatomical registration is applied to assist the surgeon when the drill is within the region on the basis of the preoperative plan. Meanwhile, it actively prevents the surgeon from cutting bone away from the defined area. The company merged to the Stanmore Implants Worldwide in 2010, and the latter released the Stanmore Sculptor Robotic Guidance Arm (RGA) System. Subsequently, MAKO Surgical obtained some confidential patents in 2013. The MAKO system is a more advanced haptically-guided one, and is currently being most commonly used for UKA, with a 20% market share for UKA in the United States [30]. MAKO system is also image-based. A preoperative CT scan is helpful in determining component size, position, and extent of bone resection. Intraoperatively, the haptic feedback system allows surgeons to safely resect bone within a pre-defined zone and prevent bone resection outside the volume [31]. The Navio system, produced by Blue Belt Technologies, now commercially available through Smith & Nephew, is a hand-held, open-platform, image-free system. It employs optically-based navigation without imaging system, to minimize the risk of radiation exposure and related cost. As a semi-autonomous system, it constantly monitors the surgeon’s performance when he or she moves the hand-held burr tool, as well as the location of the patient’s lower limb, to ensure that only the planned volume bone is removed safely and in right orientation.

### Accuracy in rUKA

UKA is a less forgiving procedure than TKA. Component malpositioning, malalignment, and ligament imbalance all contribute to a failed UKA [32, 33]. Multiple studies showed that, with robotic assistance, remarkable improvement has been made in accuracy of bone preparation, implant positioning, component alignment, and soft-tissue balance as compared with conventional techniques [10, 13, 34].

A meta-analysis assessed the accuracy of rUKA in 7 studies and indicated that robotic-assisted system in UKA could decrease the implantation errors [35]. Precision of bone preparation was evaluated in 25 cadaveric specimens on the Navio PFS system, and the final results showed that final implant position within a mean of 2 degrees rotational, and 1.3 mm translational errors of the planned target [36]. Lonner *et* *al*. compared postoperative tibial component position in 31 patients who received rUKAs with a matched group that underwent UKAs using manual instruments. The average root mean squared error (RMSE) used to evaluate postoperative component positioning was significantly improved (1.9 degrees *vs*. 3.1 degrees), with 2.6 times less variance in the robotic-assisted group (*p* = 0.02) [37]. One prospective, randomized controlled study by Bell *et al*. concluded that robotic-assisted surgery had lower RMSEs and achieved more accurate implantation of both tibial and femoral components on coronal, sagittal and axial planes (*p* < 0.01 for all parameters) [38]. Compared with manual techniques, the robotic-assisted surgery is also associated with conservative tibial resection, maintenance of posterior femoral condylar offset ratio and restitution of joint-line height, theoretically allowing reinstatement of natural kinematics and normal range of motion [39–41].

Picard *et al*. conducted a study involving 65 Navio-assisted medial UKA [42] and found that post-surgical mechanical axis alignment was within 1 degree of the intraoperative plan in 91% of cases. Batailler *et al*. [43] reported that rUKA was associated with a lower risk of postoperative limb alignment outliers. A follow-up lasting a mean time of 19.7 months showed that the rate of postoperative limb alignment outliers (± 2 degrees) was substantially lower in the robotic-assisted group than in the conventional group with both lateral UKA (26% *vs*. 61%, *p* = 0.018) and medial UKA (16% *vs*. 32%, *p* = 0.038). Plate and colleagues demonstrated that robotic-assisted systems could help the surgeon precisely reproduce plans for soft-tissue balance [44]. The authors reported that ligament balance precision was up to 0.53 mm against the preoperative protocol, with approximately 83% of cases attaining balance within 1 mm of the plan through a full range of flexion.

However, not all studies supported robotic assistance. Hansen *et al*. compared 32 Mako-assisted UKAs and 32 conventional UKAs in a follow-up that lasted for at least 2 years [45]. They reported that the robotic techniques could improve the reproduction of the preoperative femoral axis (*Pp* = 0.013), but no significant difference was found in tibial component position (*p* = 0.409). Another feasibility study by MacCallum *et al*. compared 87 rUKAs and 177 conventional UKAs in terms of tibial baseplate alignment [46]. The results showed that coronal baseplate positioning was more accurate with rUKAs (2.6 ± 1.5 degrees *vs*. 3.9 ± 2.4 degrees, *p* < 0. 0001), sagittal alignment was more accurate with conventional UKAs (4.9 ± 2.8 degrees *vs*. 2.4 ± 1.6 degrees, *p* < 0.0001). There was no difference in the percentage of implants within the safe zone between the two groups (*p* = 1.0). Bush *et al*. compared 128 consecutive medial manual UKAs performed by an experienced surgeon with published rUKAs in tibial component alignment [47]. The results showed that the percentage of preoperative target and RMSE for tibial component alignment was higher (66% *vs*. 58% / 1.48 degrees *vs*. 1.8 to 5 degrees) (Table 1).

**Table 1:** Results of accuracy in robotic-assisted UKA

Studies	System	Level of evidence	Main findings
Kwon *et al*. [62] 2019	Mako	III	During passive flexion, the mean values both before and after insertion of the implant were lower in goniometer group than in robot group.
Batailler *et* *al*. [40] 2019	Navio	III	rUKA has a lower rate of postoperative limb alignment outliers both in lateral and medial UKA, compared to conventional technique.
Iñiguez *et* *al*. [63] 2019	Navio	IV	MDFA and MPTA were significant difference with median of 1.07° *vs*. 0.12° and 1.28° *vs*. 1.3° respectively
Deese et al. [12] 2018	Mako	III	Robotic-arm assisted surgery is reported to improve the accuracy of implant placement.
Motesharei *et al*. [50] 2018	Mako	II	rUKA achieved a higher knee excursion (18.0° ± 4.9°) compared to the manual group (15.7° ± 4.1°), leading to not only better implant alignment but also some kinematic benefits to the user during walk.
Khare *et al*. [64] 2018	Navio	IV	rUKA system offers significant improvement in the femoral and tibial implant placement compared with conventional UKA system.
kayani *et al*. [49] 2018	Mako	III	rUKA improved accuracy of femoral (*p* < 0.001) and tibial (*p* < 0.001) implant positioning.
Gaudiani *et al*. [39] 2017	Mako	III	Posterior tibial slope was lower after rUKA compared to the native knee (4.91° *vs*. 2.28°, *p* < 0.0001).
Herry *et al*. [40] 2017	Navio	III	Restitution of joint-line height was improved with robotic-assisted group compared to the control group.
MacCallum *et al*. [46] 2016	Mako	III	Tibial coronal positioning was more accurate with robotic-arm-assisted (2.6° ± 1.5° *vs*. 3.9° ± 2.4°, *p* < 0.0001).
Bell *et al*. [38] 2016	Mako	II	MAKO-assisted UKA lead to improved accuracy of femoral and tibial component positioning, except for tibial coronal position.
Lonner et al. [36] 2015	Navio	IV	The image-free robotic devices achieved accurate implementation of the surgical plan with small errors in implant placement.
Mofidi *et al*. [31] 2014	Mako	III	Robotic-assisted medial UKA results in an average difference of 2.2° ± 1.7° to 3.6° ± 3.3°, inaccuracy may be attributed to suboptimal cementing technique.
Citak *et al*. [65] 2013	Mako	IV	UKA was more precise using a semi-active robotic system with dynamic bone tracking technology compared to the manual technique.
Plate *et al*. [44] 2013	Mako	III	rUKA allows ligament balancing with an accuracy of up to 0.53 mm, being 1 mm in 83% of cases.
Smith *et al*. [31] 2013	Navio	IV	The freehand sculpting tool was shown to produce accurate implant placement with small errors which are comparable to those reported by other robotic assistive devices on the market for UKA.
Karia* et al*. [66] 2013	Mako	IV	Robotic assistance enabled surgeons to achieve better precision and accuracy when positioning UKA components irrespective of their experience.
Becker *et al*. [67] 2012	KUKA	IV	The natural knee stability in antero-posterior translation and rotation can be preserved in rUKA.
Dunbar *et al*. [68] 2012	Mako	III	Implant placement errors were comparable between tactile robotics and rigid stereotactic fixation.
Pearle et al. [69] 2010	Mako	III	Haptic guidance in combination with a navigation module allows the planned and intraoperative tibio-femoral angle was within 1° and postoperative long leg axis radiographs were within 1.6° in UKA.
Lonner* et al*. [37] 2010	Mako	III	Tibial component alignment is more accurate and less variable using robotic arm assistance than manual instrumentation.
Cobb *et al*. [6] 2006	Acrobot	II	All the Acrobot cases have limb alignment in the coronal plane within 2° of the planned position, while only 40% of the conventional group achieved this level of accuracy.
Rodriguez *et* *al*. [70] 2005	Acrobot	II	All of robotic cases were implanted with tibio-femoral alignment on the coronal plane within ±2° of the planned position.

### Clinical outcomes and implant survivorship in rUKA

A meta-analysis involving 11 studies examined the clinical outcomes of rUKA and those of conventional UKA and found that rUKA could reduce the complication rate and improve knee excursion [48]. One prospective cohort study compared the early clinical outcomes in 146 patients who received medial UKAs split evenly to using either conventional devices or robotic-arm assisted devices. A less than 90-day follow-up showed that rUKA was associated with alleviated postoperative pain, reduced opiate analgesia requirements, shorter time to straight leg raise and hospital discharge, decreased physiotherapy sessions, and increased maximum knee flexion at discharge (*p* < 0.001 for all parameters) in comparison with those  receiving the conventional UKAs. Meanwhile, no difference was found in the occurrence of postoperative complications between the two groups [49]. Motesharei *et al*. conducted a prospective randomized controlled trial regarding postoperative function in 70 patients (31 receiving rUKA, and 39 receiving manual UKA) and compared them with healthy participants. Their study showed that the rUKA group accomplished better outcomes in term of kinematics from foot-strike to mid-stance than the conventional group during the postoperative 1 year [50]. Canetti *et al*. reported that rUKAs could achieve quicker return to sports than conventional UKAs (4.2 ± 1.8 months *vs*. 10.5 ± 6.7 months, *p* < 0.01) at pre-symptomatic levels [51].

As to short-term results, a retrospective study followed up 128 patients from five institutions for an average of 2.3 years and found that survivorship rate of the Navio rUKA was 99.2% [29]. Pearle and colleagues conducted a prospective multi-center study on 1135 rUKAs performed in six separate institutions and a follow-up lasting a mean time of 2.5-year [52] found that 11 knees were revised, resulting in an overall survivorship of 98.8%. The short-term survivorship of rUKA was marginally better that those reported by other large-sized follow-up studies about conventional UKA [53]. Kleeblad *et al*. followed up 432 rUKAs from four institutions in a follow-up lasting5.7-year on average and revealed a survivorship rate of 97% [54]. A recent systematic review involving 38 studies exhibited that the survivorship rate was 96%, as evidenced by a 6-year follow-up [55] . This survival rate was lower than that of the TKA survivorship in cohort (97.7%) as well as registry (96.8%) studies at mid-term follow-up [56]. These recent data showed that further long-term studies are warranted to know whether rUKA can achieve better clinical outcomes than conventional UKA, or whether the improved robotic accuracy will exerts positive influence on implant survivorship (Tables 2 and 3).

**Table 2:** Outcomes in robotic-assisted UKA

Studies	System	Level of evidence	Main findings
Kayani *et* *al*. [49] 2019	Mako	III	rUKA was associated with reduced postoperative pain, decreased opiate analgesia requirements, improved early functional rehabilitation, and shorter time to hospital discharge compared with conventional UKA.
Wong et al. [16] 2019	Mako	III	rUKA was not superior to conventional UKA in terms of functional scores, while was associated with longer operative time and cost and lower survivorship at short-term follow-up of 2 years.
Dretakis et al. [10] 2019	Mako	III	rUKA significantly improved range of motion and coronal plane alignment.
Gilmour et al. [13] 2018	Mako	II	More active patients may benefit from rUKA.
Canetti *et* *al*. [51] 2018	Navio	III	Robotic-assisted lateral UKA reduced the time to return to sports at pre-symptomatic levels when compared with conventional surgical technique (4.2 ± 1.8 months *vs*. 10.5 ± 6.7 months), with a comparable rate of return to sports (100% *vs*. 94%).
Blyth *et* *al*. [36] 2017	Mako	II	Robotic arm-assisted surgery resulted in lower median pain scores than those observed in the manual UKA group from the first postoperative day to week 8 postoperatively.
Marcovigi *et al*. [71] 2017	Mako	III	rUKA provided an improvement in terms of both clinical and technical results, and a low risk of postoperative complications.
Plate *et al*. [49] 2017	Mako	IV	Obesity had no effect on rUKA at a minimum follow-up of 24 months.
Hansen *et* *al*. [45] 2014	Mako	III	Robotic guidance did little to change clinical or radiographic outcomes, and average operative time was longer with an average of 20 min (*p* = 0.010).

**Table 3:** Survivorship in robotic-assisted UKA

Study	System	Level of evidence	Survivorship
Burger *et al*. [72] 2020	Mako	III	97.8%
Battenberg *et al*. [29] 2019	Navio	III	99.2%
Wong *et al*. [16] 2019	Mako	III	93.2%
Zambianchi *et al*. [73] 2019	Mako	IV	99.0%
Batailler *et al*. [43] 2018	Navio	III	95.0%
Kleeblad *et al*. [54] 2018	Mako	III	97.0%

### Potential downsides of robotic-assisted UKA

It is worth mentioning that rUKA is not without limitations. First, the greatest barrier for robots to go into the operating room may be its staggering initial cost. Additionally, it also imposes maintenance cost, cost of disposable elements, and education cost on surgeons and other staff. Systems that require preoperative CT scans incur additional cost of imaging examination [27]. Moschetti *et al*. showed that rUKA was more cost-effective than manual technique, only when the annual cases exceeded 94 and the failure rate was lower than 1.2% over a period of 2 years [57]. Second, advanced and novel technologies should not increase operative time at the expense of the desired outcomes. The operative time may be longer, especially when the learning curve is involved, by an average margin of 20 min [58]. However, a systematic review revealed that there was no learning curve involved for accuracy and operative time [59]. Third, current robotic systems are used to carry out a specific plan on the basis of the accurate registration data. Therefore, intraoperative insertion of percutaneous pins is required for optical tracking arrays. The intra-osseous placement of pins can theoretically cause iatrogenic complications, including pin-related periprosthetic fracture, neurovascular laceration, pin site infection or broken pins. Fourth, these systems are still unable to make creative or original decisions, or unilaterally decide how to change the surgical plan during the procedure if a new variable presents itself (*e.g*. ruptured MCL, fracture, deficient ACL). In addition, these systems will follow the designed plan and make pre-determined cuts without considering what they will cut. Therefore, the surgeon must take care of the soft tissues, or the tissues will be damaged in the planned path. Finally, 51% of papers concerning rUKA were probably industry-funded or written by authors with financial conflicts of interest, and 24% of these papers were published in journals with low impact factors or even not indexed by the Journal Citations Report. Therefore, in order to obtain more objective and accurate data and conclusions based on these papers, readers should be fully aware of possible conflicts of interests [60].

### Future robotic innovations

Current design concerning rUKA focuses on reducing outliers and increasing accuracy in radiographic outcomes. It is demonstrated that decreased revision rates and improved clinical outcomes can be accomplished by using robotic-assisted technologies. Further research effort should be directed at how to simplify the procedure and shorten the learning curve and operative time. Future innovations will likely continue to improve the preoperative plan, intraoperative sensors, and robotically-controlled instruments. About 80% of rUKA were performed in teaching hospitals and the trend would intensify in future [61]. This suggested that residents and fellows should learn to use robotic-assisted technologies earlier whenever possible during their medical education training.

## Conclusion

To date, rUKA has been demonstrated to increase accuracy and decreased outliers at the expense of increased operative time and medical cost and some short- and mid-term evidence supported its ability to improve clinical outcomes. In the future, robotics will become a valuable supplement for surgeons to simplify the operative process and make individual-specific plan. Although, more data are need to reach a definitive conclusion about the pros and cons of robotic assistance, one thing is clear: robotic-assisted operation is now being increasingly used in medical practice.

## Data Availability

The datasets used and/or analyzed in the current study are available from the corresponding author on reasonable request.

## Abbreviations

CT: Computed tomography
rUKA: Robotic-assisted unicompartmental knee arthroplasty
RMSE: Root mean squared error
THA: Total hip arthroplasty
TKA: Total knee arthroplasty
UKA: Unicompartmental knee arthroplasty
